# Quality of life among people with eye cancer: a systematic review from 2012 to 2022

**DOI:** 10.1186/s12955-023-02219-6

**Published:** 2024-01-07

**Authors:** Yonghui Huang, Yunfei Guo

**Affiliations:** Henan Provincial People’s Hospital, Henan Provincial Eye Hospital, Henan Provincial Key Medicine Laboratory of Nursing, Zhengzhou University People’s Hospital, Henan University People’s Hospital, Henan Provincial People’s Hospital, Zhengzhou, 450003 Henan China

**Keywords:** Quality of life, Retinoblastoma, Uveal melanoma, Systematic review

## Abstract

**Background:**

Eye cancer is a serious eye disease that threatens patients’ lives. In the past decade, there have been more and more studies on eye cancer. From the recently published eye cancer literature review, it can be seen that the two most popular research hotspots are retinoblastoma (RB) and uveal melanoma (UM) [1, 2]. Although several studies have assessed QOL in different types of eye cancer patients, a study that synthesizes the factors influencing QOL in eye cancer patients is yet to be undertaken. This study aimed to review and evaluate the literature related to the QOL of RB and UM survivors, and provide a synthesis of the current evidence on the impact of the two types of eye cancer on the overall QOL of patients.

**Methods:**

Eight databases (APA Psych Articles, CINAHL Complete, Health Source: Nursing/Academic Edition, MEDLINE Complete, Scopus, Cochrane Library, PubMed, and Registers (Clinicaltrials.gov.)) were searched between January 2012 and December 2022 for English, peer-reviewed quantitative original studies within this review. All publications were screened using the Preferred Reporting Items for Systematic Review and Meta-Analyses reporting guidelines. The methodological quality of the reviews was assessed using the Joanna Briggs Institute Critical Appraisal Checklists. The findings were summarised and tabulated accordingly.

**Results:**

Seventeen articles were analysed. Among them, 14 articles on patients with UM, and three articles on patients with RB using 18 different types of measurement tools were included. Eight researchers claimed that the overall QOL of patients with eye cancer was better than or similar to that of the general healthy population. However, nine studies indicated that these patients had poorer QOL than others. Many factors affect QOL, including treatment, sex, and age.

**Conclusion:**

This systematic review identified the QOL levels and several factors that influence the QOL of ocular cancer patients worldwide, due to the variability in quality of the studies, it also showed the need for further research to assess factors affecting long-term QOL outcomes in RB and UM survivors. Simultaneously, it clarified the necessity and importance of developing standardized and complete assessment tools to compare QOL in different countries. Early interventions can be developed to improve the survivors’ QOL by identifying potential deficits in specific areas.

**Supplementary Information:**

The online version contains supplementary material available at 10.1186/s12955-023-02219-6.

## Background

Quality of life (QOL) is a multidimensional construct that includes physical, emotional, functional, social, and family well-being [3]. It is considered as an important patient-reported outcome indicator that helps promote patient-doctor communication, detect symptoms, and influence medical decision making [4]. Furthermore, QOL is a highly subjective, dynamic process affected by changes in lifestyle and events [5]. Ocular tumours are serious ophthalmic diseases that threaten eyesight, QOL and morbidity, and mortality [6]. The two most prevalent eye cancers are retinoblastoma (RB) and uveal melanoma (UM) [7]. RB is a malignant eye cancer that occurs during childhood, accounting for 2–4% of childhood malignancies [1], with an incidence rate of 1/15,000–20,000 [8], and nearly 70% of cases are diagnosed within 2 years of age [9]. Globally, there are 8,000–9,000 new cases of RB being diagnosed each year [10]. Over the past 40 years, the incidence of RB in the United States (US) has been stable [11]. There is no difference in the incidence of RB according to race, sex, and region, but the survival rates of patients with RB in different countries and regions differ significantly [12]. In the US, the survival rate of RB patients reaches 95%, but in some underdeveloped countries, especially some countries in East Africa, the survival rate of patients with RB is as low as 30% [13–16]. UM is the most common intraocular malignancy in adults, affecting 2–8 cases per million people per year in Caucasians in Europe [7]. Furthermore, regardless of treatment, the 10-year metastasis-free and overall survival rates for UM-medium-sized tumours are 87% and 65%, respectively [17]. Once metastasis occurs, the average survival time of patients is 3–4 months, and the 1-year mortality rate is 80% [18]. Up to 50% of patients die from metastases within 10 years of UM diagnosis [19]. Furthermore, regardless of the treatment type, the survival rate of patients remains unchanged [20, 21]. The traditional treatments for RB and UM include enucleation surgery, chemotherapy, radiotherapy, local treatment, gene therapy and vitreous surgery [2]. Enucleation has been the standard treatment for eye cancer [22, 23], with 5-year survival rate post-surgery from 17 to 53% [24]. Although an increasing number of patients [23–25] undergo enucleation therapy, their prognosis remains poor. Approximately 35% of patients undergo enucleation [26]. Patients who are older, have more advanced tumours, and have a higher risk of metastasis often choose enucleation, which impacts health conditions [26], especially psychological trauma [12]. Moreover, enucleation is a destructive and disfiguring surgery that seriously impacts a patient’s physical and mental health [27]. In addition, postoperative vision loss can cause problems such as distance perception, and prosthetic eyes may result in irritation, discomfort, pain, and unsatisfactory appearance [28]. With the advancement of medical treatments, patients can choose between brachytherapy and proton beam radiotherapy [29, 30]. Brachytherapy is a type of radiotherapy wherein the treatment region is surrounded by a sealed radiation source. UM requires the placement of a plaque over the malignancy inside the eye using sutures from outside the eye. Although proton beam radiotherapy can preserve the eyeball with useful vision remained [21], it also increases the risk of neovascular glaucoma (44.8%), radiation retinopathy (25.4%), retinal detachment (16.8%) and local tumor recurrence (12.5%) [31].

Nearly all survivors are at an increased risk of secondary malignancies, yet concerns about the visual and genetic components of their disease remain unabated [32]. Irrespective of enucleation therapy, they are often left with monocular vision, which negatively affects motor processing, judging distance, depth perception, and increased the risk of visual dysfunction, cataracts, and severe hearing loss [33]. However, little is known about the potential impact of these long-term sequelae on the QOL of survivors. Based on these insights, QOL is a key factor in selecting an appropriate management plan for these patients [34]. Life-threatening cancer experiences and adverse effects of chemotherapy, radiotherapy, surgery, and enucleation may potentially influence the QOL of survivors [35]. Measuring QOL is critical for determining the extent to which eye cancer and its treatment impact daily life [36, 37]. Several studies have explored the QOL of patients after treatment. However, researchers hold different views on this topic. Therefore, this systematic review aimed to evaluate the literature related to the QOL of adult survivors of RB and UM, by providing a synthesis of the current evidence investigating the impact of these two types of eye cancer on the overall QOL of patients. Furthermore, this review can provide advice on further studies on these patients and, measures to improve their QOL.

## Methods

The Preferred Reporting Items for Systematic Review and Meta-Analyses (PRISMA) reporting guidelines were followed [38]. At the beginning of the study, a protocol was developed and registered with PROSPERO (CRD42022283279).

### Search strategy

The search strategy was developed by the authors with the support of two librarians at Munster Technological University. The search strategy was tested using an iterative process before finalisation (Table 1). Eight databases were searched: APA Psych Articles, CINAHL Complete, Health Source: Nursing/Academic Edition, MEDLINE Complete, Scopus, Cochrane Library, PubMed, and Registers (ClinicalTrials.gov.). The reference lists of selected articles were checked for potentially relevant articles. The initial search was conducted on 19 May,2022, and updated on 31 December, 2022.

**Table 1:** Search strategy table

Date	2022/5/19	
**Research Topic**	The quality of life among people with eye cancer	
**Search Strategy**	**Keywords/concepts**	**Synonyms/alternative terminology**
	**Ophthalmic**	“Ophthalmic*” OR “eye” OR “ocular” OR “optic*”
	**Cancer**	“Melanoma” OR “cancer” OR “tumor” OR “malignan*” OR “oncolog*” OR “neoplasm*” OR “Retinoblastoma” OR “uveal melanoma” OR “UM” OR “RB”
	**Quality of life**	“Quality of life” OR “QoL”
**Limits and Type of material required**	2012–2022	
	English language	
	Adults over the age of 18 years	
	Peer-reviewed scholarly articles	
**Databases searched**	APA Psych articles, Academic Search Complete, CINAHL Complete, MEDLINE Complete, Health Source: Nursing/Academic Edition, PubMed, Scopus, ScienceDirect, Cochrane Library, and Clinicaltrials.gov.	

### Eligibility criteria

Studies that investigated the QOL of patients with UM or RB only (> 18 years old), regardless of whether they were living at home or in any care setting were eligible for inclusion. Additional inclusion criteria were quantitative studies published in English between January 2012 and December 2022.

### Study selection

All records were imported into Endnote X9 and duplicates were removed. Subsequently, the records were exported to Rayyan [39], where two reviewers (Y.H. and C.O.B.) independently completed the title and abstract screening based on the eligibility criteria outlined above. Following this step, the full-text articles were identified and read by the reviewers to determine their final eligibility. All eligible patients were retrieved and the full text was added to Rayyan [39]. Two reviewers (Y.H. and C.O.B.) read each article to determine eligibility for inclusion. Disagreement between the two reviewers was resolved by a third reviewer (D.L.).

### Data extraction and risk of bias assessment

Two reviewers (Y.H. and Y.G.) independently extracted the data into an Excel file. The extracted data consisted of the study and participants’ characteristics (Table 2). The Joanna Briggs Institute critical appraisal checklists (for cross-sectional and cohort studies, as appropriate) were used to evaluate the risk of bias and quality of each article. This review was completed independently by two authors (Y.H. and C.O.B).

**Table 2:** Summary of the QOL outcomes by treatment modality

Author(s) (Year)	Treatment	a) Sample size(*n*) b) M/F	a) Outcome Indicator(s) b) Outcome Measurement Tool(s)	Survey Follow-Up		Difference in QOL	Study results
Klingenstein et al. (2013) [43]	CyberKnife (*n* = 91)	a) 91 b) 48/43	a) QOL b) SF-12	4–15 months		Yes at some aspects	Physical functioning and role physical decreased significantly after CyberKnife radiosurgery, while mental health improved (*P* = 0.007, *P* < 0.0001 and *P* = 0.023). Mental health and social functioning were significantly increased in the non-glaucoma group (*P* = 0.0003 and 0.026), and mental health was higher than in glaucoma patients (*P* = 0.02). At the second follow-up, patients with higher best corrected visual acuity had significantly higher PF and role physical (*P* = 0.02). Role physical was decreased in patients with best corrected visual acuity < 0.5 log (MAR) (*P* = 0.013). Viability was significantly increased in patients with preserved best corrected visual acuity (*P* = 0.031).
Wiley et al. (2013) [47]	Enucleation (*n* = 16), brachytherapy(*n* = 79), resection (*n* = 35), proton beam therapy (*n* = 3)	a) 99 b) Not mentioned	a) the needs, vision-targeted health, depression, fears b) CNQ-SF, NEI-VFQ-25, CES-D, CARS	Within the prior 5 years (M = 2.05)		Yes	Although concern about cancer recurrence was elevated, QOL was better than in other oncology samples and comparable with healthy samples on some outcomes. Enucleation was associated with poorer vision-specific QOL, and the presence of comorbidities was associated with poorer vision-specific QOL, depressive symptoms, and worry about cancer recurrence (all *P* < 0.05). More reported unmet cancer needs were associated with worse vision-specific QOL, depressive symptoms, and more worry about recurrence (all *P*​<0.05)
Ford et al. (2015) [16]	Adult RB survivors (*n* = 470) , the Childhood Cancer Survivor Study (*n* = 2,820)	a) 470 b) 245/225	a) Psychological distress, subjective distress, the extent to perceive personal benefits, fear, facial appearance satisfaction b) BSI-18, IES, PTGI, Fear of Recurrence Questionnaire, Self-report chronic conditions and satisfaction with facial expressions	/		No	Compared with the Childhood Cancer Survivor Study siblings, the depression, somatization, distress, or anxiety rates of RB survivors did not increase significantly. Among survivors, chronic diseases did not increase the possibility of psychological problems. Compared with unilateral RB survivors, bilateral RB survivors are more likely to experience the fear of cancer recurrence and fear that their children will be diagnosed with RB.
Klingenstein et al. (2015) [37]	Enucleation(*n* = 32), stereotactic radiosurgery(*n* = 48) , age-matched control group(*n* = 35)	a) 115 b) 60/55	a) QOL b) SF-12	3-year follow-up. 37.9 months After stereotactic radiosurgery versus 42.9 months after enucleation	No between CK and normal group, yes between enucleation and general people at physical functioning, role physical,RE		There was no significant difference in QOL between patients treated by stereotactic radiosurgery and the age-matched control group. After enucleation, patients presented significantly lower values in physical functioning, role physical, and QOL was not significantly different between patients treated with stereotactic radiosurgery and age-matched controls. However, physical functioning, role physical, and role emotional values ​​were significantly lower in the enucleation group compared with radiosurgery and controls. At mid-term follow-up, there were no significant differences between stereotactic radiosurgery, enucleation, and the control group in terms of general health, vitality, social functioning, or mental health. In multivariate analysis, the number of comorbidities had a significant effect on QOL.
Hope-Stone et al. (2016) [40]	Enucleation (*n* = 117), brachytherapy: ruthenium plaque (*n* = 174), proton beam radiotherapy (*n* = 72), local resection (*n* = 15), endoresection (*n* = 21), photo dynamic therapy (*n* = 10), transpupillary thermal therapy (*n* = 2)	a) 411 b) 218/193	a) Anxiety and depression, HRQOL b) HADS, FACT-G	6 months, 1 year, and 2 years after treatment		No	In terms of QOL and depression, patients are close to or better than the standard value at all time points, but younger women were more anxious than normative. Results did not differ at any time depending on whether patients were enucleated or not. There were no differences in outcomes at any point depending on whether patients were enucleated or not. Patients with monosomy 3 showed more depressed mood at 6 months, 1 year, and 2 years after treatment (*P* < 0.05).
Damato et al. (2018) [30]	Enucleation (*n* = 442) , Radiotherapy (*n* = 1154 (ruthenium plaque radiotherapy (*n* = 730) and proton beam radiotherapy (*n* = 424))	a) 1596 b) 825/771	a) QOL, anxiety and depression, HRQOL b) EORTC QLQ-OPT30, HADS, FACT-G	6 months, 1 year, and 2 years after treatment		Yes	Enucleation was associated with males, older age, larger tumor diameter, monosomy 3, depression, and decreased physical and mental health. QOL was better in patients who underwent radiation therapy than enucleation, but worse than in the general population. Anxiety scores were lower than those of the general population in the UK. Enucleation patients had better social functioning and poorer emotional functioning than the general population, but better physical functioning than US cancer patients, receiving radiation treatment had better overall FACT-G scores than cancer patients, with poorer QOL independent of treatment modality. Patients receiving radiation were less likely to complain of ocular discomfort (gritty, itching, tearing, discharge), and less concerned about their appearance and the risk of losing their eyes. The radiation therapy group also reported fewer visual difficulties (driving in the dark, pouring, walking in crowds, seeing steps, walking on uneven ground, judging distances).
Frenkel et al. (2018) [44]	Brachytherapy (*n* = 198), enucleation (*n* = 19), local resection (*n* = 10), proton beam (*n* = 6)	a) 233 b) 102/131	a) QOL b) EORTC QLQ C30, QLQ-OPT30.	2 weeks to 295 months (76 ± 62.4 month)		Yes in some aspects, no overall	There were no statistically significant differences in general QOL scores for different types of initial treatment. Better QOL was associated with tumors that did not involve the ciliary body and better corrected visual acuity. However, patients who underwent enucleation had lower eye-related QOL compared with those who received brachytherapy and described body image-related issues, but cosmetic concern was not significantly different between treatment groups.
Friedman et al. (2018) [33]	Radiation or Chemotherapy or enucleation (*n* = 470)	a) 470 b) 225/245	a) Vision-targeted health b) NEI-VFQ-25	/		Yes in some aspects, no overall	Overall QOL was not bad, with an overall mean VFQ score of 81.1 for all survivors. Previous radiation therapy was not associated with decreased VFQ, but it was associated with certain subregions of visual function. Bilateral enucleation is associated with lower QOL, unilateral disease also affects QOL (*P* < 0.001), and enucleation also affects QOL (*P* = 0.002). Vision-related QOL scores are generally good (unilateral 91.4, bilateral72.3).
van Beek et al. (2018) [45]	Fractionated stereotactic radiation therapy(*n* = 65) ,. enucleation (*n* = 48)	a) 113 b) 60/53	a) Anxiety, subjective distress, QOL, vision-targeted health b) STAI, IES, EORTC-QLQ-C30, NEI-VFQ-25	Before treatment and 2, 6, 12, 24, 36 and 48 months after treatment.		Yes at short-term, no at long-term	The overall global health score was 76.4. The role functioning score from 2 months to 3 years after treatment, the QOL in the enucleation group decreased (*P* = 0.012), and that in the fractionated stereotactic radiation therapy group increased (*P* = 0.005), 4 years after treatment, there was no significant difference in QOL scores between the two treatment groups. Patients with metastases were more anxious than those without. Subjective pain in patients with metastases compared with those without metastases at baseline (*P* = 0.037). Increases in overall pain (*P* = 0.023) and emotional functioning (*P* < 0.001) were observed after 1 year of treatment. Decreased physical functioning (*P* = 0.035), insomnia (*P* < 0.001) and anxiety (*P* < 0.001) from pre-treatment to 2 years post-treatment. Patients with enucleation had worse peripheral vision observed up to 3 years after treatment (*P* = 0.026).
Barker et al. (2019) [17]	Radiotherapy (*n* = 201)	a) 201 b) 111/90	a) QOL b) EORTC QLQ-C30, QLQ-OPT30	After diagnosis and prior to treatment with radiotherapy.		No	Overall QOL was good, with the most common severe QOL concern being worry about disease recurrence (41%). The most common ophthalmic symptoms were visual impairment (81%) and eye irritation (66%). Overall QOL was related to demographics, but not strongly (*P* < 0.01, r = 0.14), and patients with advanced disease had poorer QOL after initial diagnosis.
Damato et al. (2019) [41]	Enucleation (*n* = 442) , radiotherapy (*n* = 1154, ruthenium plaque brachytherapy (*n* = 730), proton beam radiotherapy (*n* = 424))	a) 1596 b) 825/771	a) QOL, anxiety and depression, HRQOL b) EORTC QLQ-OPT30, HADS, FACT-G	6 months, 1 year, and 2 years after treatment.		Yes	Self-reported QOL decreased with both groups. Treatment modality is not related to QOL level. Overall anxiety tended to decrease and depression increased, especially after enucleation. Emotional health improved after treatment, while functional and physical health decreased after enucleation but improved after radiotherapy. The overall FACT-G score increased in the radiotherapy group and decreased in the enucleation group. Depression had nothing to do with treatment, but was related to unemployment, which occurred more among enucleation treatment. Over time, visual difficulties decreased after enucleation but increased in patients who received radiation therapy, and concerns about metastasis, health loss, and tumor recurrence decreased in both groups.
Hope-Stone et al. (2019) [28]	Enucleation (*n* = 66) , proton beam radiotherapy(*n* = 49)	a) 115 b) 56/59	a) QOL b) EORTC QLQ OPT30	6-, 12-, and 24-months following diagnosis.		Yes	Patients treated with enucleation having more functional problems at 6 months, which were resolved at 12 and 24 months (*P* = 0.020). Central and peripheral visual impairment (*P* = 0.009) and dyslexia (*P* = 0.002) were more severe in proton beam radiotherapy patients within 24 months. But treatment did not affect driving (*P* = 0.694), eye irritation (*P* = 0.281), headache (*P* = 0.640), cosmetic problems (*P* = 0.187) or fear of recurrence (*P* = 0.899).
Scannell et al. (2019) [5]	PR (*n* = 103) , enucleation (*n* = 35)	a) 138 b) 78/60	a) QOL b) EORTC QLQ-C30, QLQ-OPT30	/		No	There was no significant difference in QOL score between the treatment groups. 32% of the patients were worried about tumour recurrence in other parts of the body. The role functioning score of the brachytherapy group was significantly higher (*P* = 0.030). Patients with enucleation are more prone to appearance problems (*P* < 0.0005). Younger patients (12–54 years old) are more likely to report headaches (*P* < 0.0005) and dyslexia (*P* = 0.042), and the cognitive function score (*P* = 0.003) is lower than patients ≥ 55 years of age.
Feng et al. (2020) [49]	Enucleation (*n* = 66) , healthy adults(*n* = 66)	a) 132 b) 53/79	a) HRQOL, anxiety and depression, fear, facial appearance dissatisfaction b) Chinese version of the SF-36, HADS, FoP-Q-SF, NPSS-F	/		No	Adult RB survivors did not have significantly higher rates of depression and anxiety compared with the control group, and they experienced a relatively good QOL. RB survivors were more likely to worry about their facial appearance (*P* < 0.001). Radiotherapy was not the factor affecting satisfaction with facial appearance (*P* = 0.214). Females were more likely to be influenced by the disease (*P* = 0.031) and worry about their appearance (*P* = 0.041).
Brown et al. (2021) [42]	Enucleation(*n* = 177), plaque radiotherapy (*n* = 304), resection (*n* = 35), proton beam radiotherapy (*n* = 155) other (*n* = 22)	a) 824 b) 435/389	a) QOL, anxiety and depression, HRQOL b) EORTC QLQ-OPT 30, HADS, FACT-G	6, 12 and 24 months after diagnosis		No	Overall QOL was good, with anxiety and depression similar to the UK population, and QOL similar to the Australian population. Single predictor analyses showed 6-month depression and poorer functional QOL predicting mortality, as did 6–12 month increases in anxiety and decreases in physical and functional QOL. Multivariate analyses showed independent prediction by 6-month depression and decreasing QOL over 6–12 months and 12–24 months.
Gollrad et al. (2021) [46]	Surgery + Radiotherapy (*n* = 131)	a) 131 b) 66/65	a) QOL, anxiety b) EORTC QLQ-C30, QLQ-OPT30, GAD-7	Before clip-surgery, between clip-surgery and proton therapy, after proton therapy, and three months after treatment		Yes at short-term, no at long-term	Global QOL was similar to that in Germany before treatment, and returned to baseline levels three months after treatment. Compared with clip-surgery and three months after treatment, there was no change, but three months after treatment had more eye-specific symptoms than clip-surgery (*P* < 0.001). Compared with before treatment, physical functioning and role functioning still deteriorated significantly (*P* < 0.001), social functioning and emotional functioning improved over time. QOL and an increase in eye-related symptoms, because of the clip surgery (clip-surgery-between clip-surgery and proton therapy). After treatment completion, global QOL improved gradually, and none of the eye-related symptoms significantly deteriorated over the course of proton therapy.
Gollrad et al. (2022) [48]	PBT (*n* = 108), observation (*n* = 61), intravitreal injections (*n* = 17), intraocular surgical procedure (*n* = 30).	a) 108 b) 52/56	a) QOL b) EORTC QLQ-C30, QLQ-OPT30	At baseline, and at 3 and 12 months		Yes at short-term, no at long-term	All have a stable global health scores in the short term. In the latter group, several QOL items significantly declined after the 3-month adjuvant interval, but they partially recovered at the 12-month follow-up. In all adjuvant-intervention groups, global QOL scores returned to baseline levels at 12 months. Visual acuity correlates with QOL.
RB = retinoblastoma; UM = uveal melanoma; QOL = Quality of life; HRQOL = health related quality of life; SF-12 = 12-Item Short Form Survey; SF-36 = 36-Item Short Form Survey; EORTC = the European Organization for Research and Treatment of Cancer; QLQ-OPT30 = Quality of Life Questionnaire Ophthalmic Module; QLQ-C30 = The Core Quality of Life questionnaire; CNQ-SF = the Cancer Needs Questionnaire-Short Form;NEI-VFQ-25 = 25-item National Eye Institute Visual Function Questionnaire; CES-D = The Center for Epidemiologic Studies Depression scale; CARS = The Concern about Recurrence scale;BSI-18 = The Brief Symptom Inventory-18; IES = The Impact of Events Scale; PTGI = The Post-Traumatic Growth Inventory; HADS = Hospital Anxiety and Depression Scale; FACT-G = the Functional Assessment of Cancer Therapy-General; VFQ = Visual Function Questionnaire; STAI = The State-Trait Anxiety Inventory; FOP-Q-SF = Fear of Progression Questionnaire-Short Form; NPSS-F = the Negative Physical Self Scale-Facial Appearance Concern; GAD-7 = General Anxiety Disorder-7.							

### Data synthesis and analysis

A narrative synthesis of findings of all studies is presented below.

## Results

**Fig. 1:**
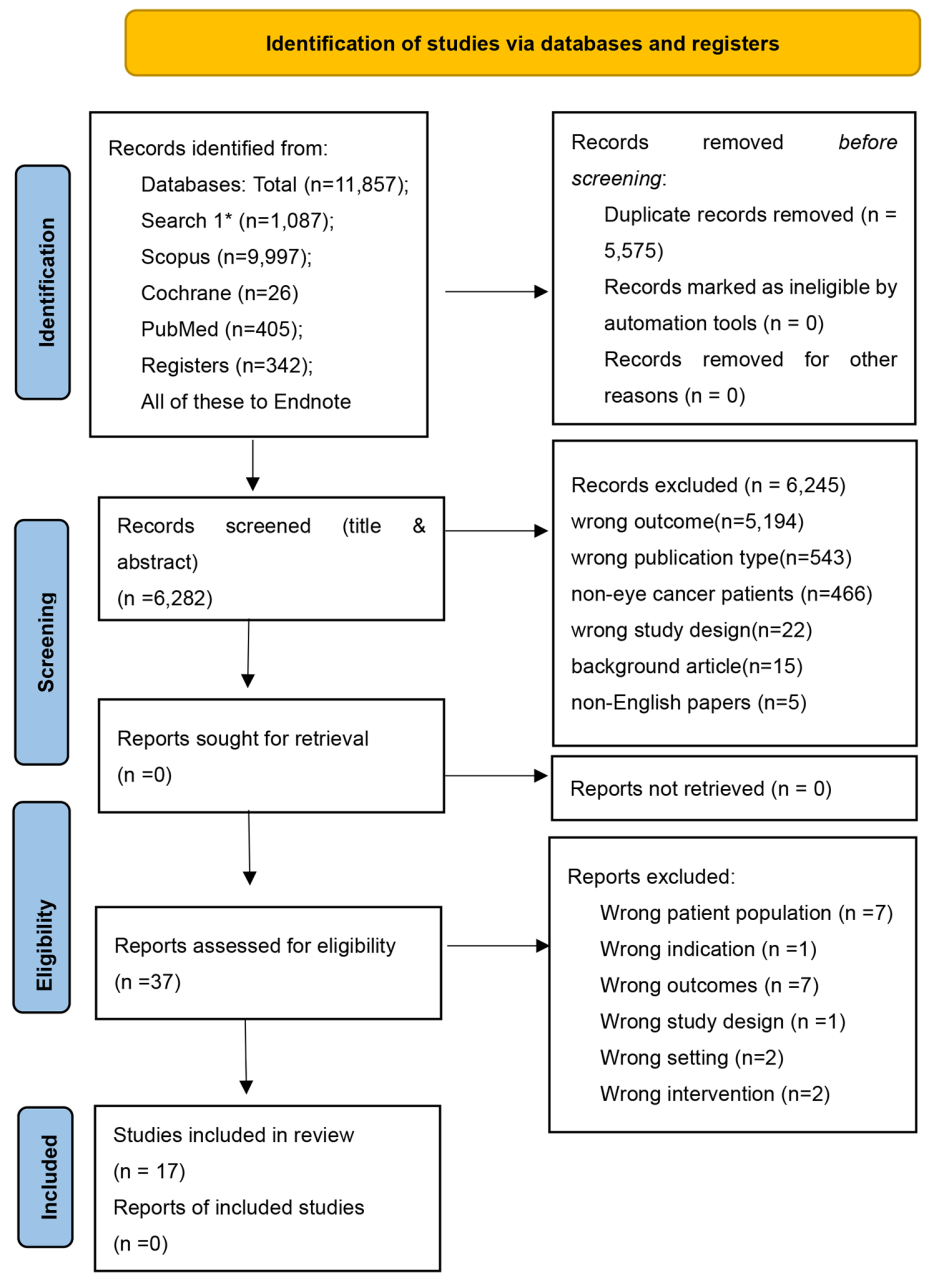
PRISMA flow diagram. Search 1* = APA Psych Articles, CINAHL Complete, Health Source: Nursing/Academic Edition, MEDLINE Complete. Total: 11,857 (19/05/2022)

The original search identified 11,857 articles. After removing duplicates, 6,282 titles and abstracts were screened for eligibility, with 37 remaining for a full-text review. Finally, 17 studies were included in the systematic review (Fig. 1).

### Study characteristics

Most of the included studies (15), investigated UM [5, 17, 28, 30, 37, 40–48] with three studies on RB [16, 33, 49]. Among the included articles, one [49] study was carried out in a low- or middle-income country (China) and 16 studies were conducted in high-income countries: the US (9) [16, 17, 28, 30, 33, 40–42, 47], Germany (4) [37, 43, 46, 48], Ireland (1) [5], Israel (1) [44], and the Netherlands (1) [45]. In relation to study design, there are ten cohort studies [28, 30, 33, 37, 40, 41, 44–46, 48], five cross-sectional studies [5, 16, 17, 47, 49], and two longitudinal studies [42, 43]. The characteristics of the included studies are summarised in Table 2.

### Patient reported outcomes of QOL - assessment tools

Eighteen different assessment tools were used to measure patients’ QOL across all included studies (Table 2). Eight articles used more than two different tools to measure outcomes [16, 30, 41, 42, 45–47, 49]. The most commonly used tools were Quality of Life Questionnaire Ophthalmic Module (QLQ-OPT30, measured the QOL for UM patients) [5, 17, 28, 30, 41, 42, 44, 46] and the Core Quality of Life questionnaire (QLQ-C30, measured the QOL for patients with all kinds of cancer) [5, 17, 44–46] authored by the European Organization for Research and Treatment of Cancer (EORTC); the Functional Assessment of Cancer Therapy-General (FACT-G, measured physical, social, emotional, and functional well-being domains of QOL in patients with cancer) [30, 40–42], National Eye Institute Visual Function Questionnaire (NEI-VFQ, measured the dimensions of self-reported vision-targeted health status that are most important for patients with chronic eye diseases) [33, 45, 47], Hospital Anxiety and Depression Scale (HADS, measure anxiety and depression in a general medical population) [30, 40–42, 49] and 12-Item Short Form Survey (SF-12, assessed the impact of health on an individual’s everyday life) [37, 43].

### The overall QOL level

The findings reported in the included studies indicated variations in the QOL of eye cancer survivors across studies. Six studies showed that the overall QOL scores of patients with UM or RB were higher than or similar to those of the healthy general population [5, 16, 17, 40, 42, 49]. However, 11 studies indicated that these patients had a poorer QOL than the healthy general population [28, 30, 33, 37, 41, 43–48]. Three studies investigated QOL in patients with RB [16, 33, 49], two of which concluded that the overall QOL was better than that in non-cancer controls [16, 49], and one concluded that QOL was affected by vision-targeted HRQOL in some aspects; for example, subdomains of visual function such as peripheral vision, especially in bilateral survivors of disease (*P* < 0.001) or with enucleation (*P* = 0.002) [33]. There were 14 articles investigating the overall QOL of UM patients, 4 out of 14 reported that there is no difference between QOL of these patients and the general population [5, 17, 40, 42], 6 out of 14 reported that the QOL was affected and decreased [30, 37, 41, 43, 44, 47], and 4 out of 14 indicated that the QOL decreased first and then there was no difference with time [28, 45, 46, 48]. Six articles [28, 37, 40, 45, 46, 48] noted poorer QOL at the start of treatment but at the 3 [46], 6 [28, 40],12 [28, 40, 48], 24 [28, 40], 36 [37] and 48 months [45] follow-ups, there were no differences when compared to the general healthy population. In contrast, Frenkel et al. [44] suggested that treatment has a greater impact on the patient’s life during the first 2 years than later, however this was not statistically significant (*P* = 0.073); the other QOL domains were not related to the post-treatment duration. Only in the cohorts of van Beek et al. [45] and Gollrad et al. [46, 48], pretreatment status was assessed. In addition, van Beek et al. [45] consistently reported that anxiety problems improved in patients with UM within 2 years after primary treatment compared to pretreatment assessments [45]. They also found that from 2 months to 3 years after treatment, the role functioning score increased (*P* = 0.005) in patients who had undergone irradiation, and there was a decline in physical functioning (*P* = 0.035), insomnia (*P* < 0.001), and anxiety (*P* < 0.001), whereas increases in overall pain (*P* = 0.023) and emotional functioning (*P* < 0.001) were observed 1 year posttreatment [45]. Decrease in physical functioning (*P* = 0.007), role physical functioning (*P* < 0.0001), and improvement in mental health (*P* = 0.023) at 1- and 2-year follow-ups after stereotaxic CyberKnife radiosurgery were demonstrated by Klingenstein et al. [43]. In Gollrad et al. [46], subsequent emotional and social functioning improvement after treatment were similar to those of previous studies that improved overtime.

Overall, RB patients have better QOL scores than UM patients, with two out of three studies [15, 49] showing that RB patients had scores similar to general population, thereby reporting fewer QOL-related problems. However, the QOL in patients with UM is variable, with inconsistent results across studies. Only 6 of 14 studies [5, 17, 28, 37, 40, 42] indicated that the QOL was not different or better than that of the general population.

### Age and sex differences in QOL

Ten studies reported the effects of age and sex on QOL [5, 17, 30, 33, 37, 40, 43, 46, 47, 49]. Younger female patients were more likely to have problems that affected their QOL or sub-items [5, 17, 37, 43, 46, 49], and higher anxiety [30, 40, 46] (*P* < 0.001). These differences can be classified into physical and mental outcomes. As for physical outcomes, Gollrad et al. [46] found that younger patients’ global health was worse (after the clip surgery procedure; *P* = 0.021), with more headaches (pretreatment and final assessment; *P* < 0.04, post-proton treatment (*P* = 0.006). Scannell et al. [5] also found that younger (12–54 years old) female patients were more likely to report headaches (*P* < 0.0005) and reading difficulties (*P* = 0.042). Similarly, Klingenstein et al. [37] found differences in physical functioning according to age and sex (all *P* < 0.05). Barker et al. [17] also found that age (*P* < 0.01) and sex (*P* = 0.03) influenced on physical functioning scores. Klingenstein et al. [43] reported that physical functioning decreased significantly in male survivors (*P* = 0.030), and role physical functioning decreased significantly in female survivors (*P* = 0.017). Damato et al. [30] also reported that women’s physical well-being improved in females. However, Wiley et al. [47] found that sex and age were not associated with role difficulties (*P* > 0.05). Friedman et al. [33] showed that age and sex did not affect NEI-VFQ-25 scores. Regarding mental outcomes, Gollrad et al. [46] found that only female patients had a significant decrease in QOL between pretreatment and post-surgical assessment (*P* < 0.001), with lower emotional functioning at all valuation points (pretreatment: *P* = 0.042, post-surgical assessment: *P* = 0.037,post-proton treatment: *P* = 0.014, final assessment: *P* = 0.004), and higher anxiety (*P* < 0.001). Barker et al. [17] also found that role functioning (*P* = 0.01) and emotional functioning (*P* < 0.01) were related to sex. Similarly, Feng et al. [49] found that women were more susceptible to the disease (*P* = 0.031) and more concerned about their appearance (*P* = 0.041). Further, Klingenstein et al. [43] reported that mental health in male survivors improved after 1 year (*P* = 0.042), but there was no difference at 2 years (*P* = 0.16). Hope-Stone et al. [40] assessed anxiety levels 2 years after treatment and found that younger patients were more anxious (*P* < 0.01). However, Wiley et al. [47] found that sex and age were not associated with mental health or fear of recurrence (*P* > 0.05). Similarly, Damato et al. [30] mentioned that women were more anxious but their emotional well-being was better. Klingenstein et al. [37] found that there were differences in role-emotional scores by age and sex (all *P* < 0.05), and in mental health scores by sex (*P* = 0.021), but they did not mention the specific differences.

### Treatment differences in QOL

Twelve articles [5, 28, 30, 33, 37, 40–42, 44, 45, 47, 48] explored the relationship between the treatment received and subsequent QOL. Table 2 summarises the QOL outcomes reported by treatment in each included study. Among 12 comparative studies, three reported no overall difference in QOL between the different types of treatments [5, 41, 42, 47]. Three of 12 studies reported a significant difference in at least one QOL subdomain for one treatment modality compared to other modalities [28, 30, 33, 37]. Three of the 12 studies described no overall differences in long-term follow-up but noted some significant differences in QOL in specific functional domains (e.g. vision problems) [44, 45, 48], anxiety [40, 45], depression [40, 45], and short-term physical functioning [45].

Ten studies indicated that enucleation affected QOL [5, 28, 30, 37, 40–42, 44, 45, 47]. Klingenstein et al. [37] compared the QOL of patients with UM treated with CyberKnife or enucleation with that of the general population and found that radiation did not affect QOL, whereas enucleation decreased physical and emotional parameters, especially those associated with physical functioning (*P* = 0.0063), emotional role (*P* = 0.012), physical role (*P* = 0.043) and body pain (*P* = 0.037). Hope-Stone et al. [28] reported that patients with UM that had undergone enucleation faced more and worse functional problems at 6 months, which diminished at 12 and 24 months (*P* = 0.020); however, QOL did not differ in terms of driving difficulties (*P* = 0.694), eye irritation (*P* = 0.281), headaches (*P* = 0.640), appearance problems (*P* = 0.187) and fear of recurrence (*P* = 0.899). Damato et al. [30] reported that patients who underwent primary enucleation had worse QOL. However, as mentioned in their subsequent study [41], QOL among the different treatment modalities did not differ, and patients’ QOL recovered to a level similar to that of the general population within 6 months, which is in agreement with Hope-Stone et al. [40], who reported that there was no difference in any measure of QOL, regardless of enucleation. However, Scannell et al. [5] and van Beek et al. [45] reported no difference in QOL between patients who underwent enucleation and those who did not (89.3 vs. 89.2, respectively; 78.8 [fractionated stereotactic radiation therapy] vs. 78.3 [enucleation]). Frenkel et al. [44] also demonstrated that the general QOL did not differs at initial treatment, but those who underwent enucleation had a lower eye-related QOL than those who received brachytherapy (*P* = 0.019) which is in line with Wiley et al. [47], who claimed that compared with patients receiving brachytherapy, patients undergoing enucleation had more role difficulties. Brown et al. [42] found that, regardless of the treatment modality, emotional QOL increased in patients with UM at 6, 12, and 24 months after diagnosis.

## Discussion

Despite the differences in QOL among patients with eye cancer, insufficient research has been conducted, and only 17 relevant studies have been published in past ten years. The included studies used 18 different kinds of questionnaires, and the proportion of UM-specific assessment tools (OPT-30) among patients reached 57.14% (8 of 14) [5, 16, 28, 30, 41, 42, 44, 46]. In addition to the core QOL questionnaire, 9 of 17 studies [16, 30, 40–42, 45–47, 49] addressed important aspects such as anxiety, depression, fears, concerns, and financial burden. However, with follow-up from baseline [45, 46, 48] to a maximum of 295 months [44], it was reported that treatment did not impact the survival rate of patients, but the QOL differed over time; therefore, a longitudinal assessment using the same measurement of QOL is essential [4, 50] and has become an important focus of clinical decision-making [5, 28, 37, 41–46]. Researchers have also noted that QOL is affected by various factors, such as age, sex, treatment, and general health.

Many factors could have caused bias among the 17 articles. As for the sampling distribution firstly, because of age, sex, social factors, tumour size, and location, it is immoral to assign patients to randomised controlled trials [5, 28, 30, 41, 45]. Secondly, as shown in Table 2, although the proportion of men and women included is roughly equal, the sample size varies greatly, ranging from 91 [43] to 1596 [30, 41] people. Some studies with small samples may lack the statistical significance of the data. However, owing to the low incidence and high mortality of this cancer, and the differences in follow-up time between different studies also pose challenges to the data collection of large-sample and long-term studies, therefore in the absence of sufficient time, researchers will obtain a small sample size [28, 44, 48]. Thirdly, only three articles had baseline data [45, 46, 48], which meant that the other studies were unable to have internal comparisons, because they did not know the original status of these patients. Fourthly, fewer enucleation patients were involved, which may be because patients received radiotherapy need more regular follow-up treatment, therefore easier for recruitment [30]. Similarly, Ford et al. [16] excluded disabled survivors, which is a selection bias. Simultaneously, Hope-stone et al.’s [28] cohort had a high loss of follow-up rate of up to 40.7%, and the other research conducted by them [40] also had a loss rate of 17%, which is similar to Feng. et al. [49], whose loss of follow-up was 30.2%, which means that some patients failed to complete the entire follow-up period and withdraw from the study, resulting in higher loss to follow-up rate, the result may not represent the real conditions of the survivors. Moreover, the differences in outcomes between countries may be related to different medical and social support systems, for example, the included studies come from 9 cities in 6 different countries, with different inclusion and exclusion criteria and regional differences, except for Klingenstein et al. [37] and Feng et al. [49] who used quota sampling, the remaining 15 articles used convenience sampling to recruit the target samples. Additionally, only one article from a developing country was identified [48]. One reason for this may be the higher incidence of eye cancer in Europe and the US, which means that more people are affected; moreover, these countries have sufficient experience and available tools compared to other countries. However, because of the lack of advanced medical conditions in developing countries, the long-term survival rate of patients with eye cancer remains unchanged, with shorter survival times [49]. All the above showed that the samples may not well represent the overall QOL worldwide. Sampling variability may be related to the location of the participants and sampling error. Therefore, it would be beneficial to seek out and include populations from different regions in future studies, which may clarify the existing inconsistencies.

As for the Publication bias, first, among the included articles, 5 of 17 were published in two journals, including three in Ocul Oncol Pathol [5,16.41] and two in Acta Ophthalmol [44, 45]. second, as for the authors, Hope-stone, Damato, Brown et al. were in the same team, and published five articles in 2016, 2018, 2019, and 2021 with patients treated in different periods and measured with different questionnaires at different time [28, 30, 40–42]. Similarly, Gollrad et al. [46, 48] published two articles in 2021 and 2022 with different target population at different periods. Klingenstein et al. [37,53] also published two articles in 2013 and 2015 using the same questionnaire but measured at different time points, but part of the data was obtained by extending the follow-up time based on the data in 2013. Third, positive results and large sample results are easier to publish. Among the studies included in this review, 11 (i.e., positive results) considered QOL to be worse than the general population, accounting for 64.71%. Luckly, we initially searched the Clinicaltrials.gov which may include some grey literature to ensure comprehensiveness. Forth, only seven research articles (41.18%) [17, 30, 33, 44–47] mentioned fund support but with no conflict of interest, which was also an indicator to evaluate publication bias. Fifth, we excluded non-English articles due to language barriers, which may lead to language bias. All of the above have reduced publication bias to a certain extent.

The results also lacked comparative and normative data for healthy controls. Without a standardised questionnaire, the results varied significantly. Furthermore, the included studies used a variety of methods, with different follow-up periods, sample sizes, and demographics, which resulted in heterogeneity, all of which make it difficult to generalise the findings from one study to larger patient populations. Compared to patients with UM, RB patients experience relatively fewer functional problems, and less anxiety and depression, which may be because RB survivors survive longer than patients with UM, and RB occurs more frequently in children; when they grow up, they usually cherish life more with high resilience than healthy people [16, 49].

### Limitations and next-step recommendations

This systematic review had some limitations. First, there were no randomised controlled trials in the included studies and only patients with RB and UM were included, which may not fully represent eye cancer patients. Second, because of the heterogeneity of the treatments, assessment tools and different follow-up times in each study, it was difficult to effectively compare the data included in this review. Therefore, it is necessary to develop a standardised QOL assessment tool for patients with eye cancer. Further, it is recommended that more attention be paid to developing a specific standard assessment tool, focusing on the QOL of eye cancer survivors in low- or middle-income countries and taking measures to improve their QOL.

## Conclusion

Through this review, it is found that there are differences in the current QOL of patients with eye cancer, with anxiety and depression occurring more frequently. However, it is difficult to assess QOL accurately because of the differences in treatment, assessment tools, and follow-up times. Therefore, it is necessary to develop a standardised QOL assessment tool and a follow-up protocol. It is worth noting that more attention should be paid to developing countries, whose mortality rates are high, to improve their QOL.

### Electronic supplementary material

Below is the link to the electronic supplementary material.

Supplementary Material 1

## Data Availability

Not applicable.
