# Supplementary material for: Quality of life among people with eye cancer: a systematic review from 2012 to 2022
Source: Health Qual Life Outcomes. 2024 Jan 7;22:3. doi: 10.1186/s12955-023-02219-6 (PMC10773080; doi:10.1186/s12955-023-02219-6)
Supplement: Supplementary file 1 — Supplementary Material 1 [file 12955_2023_2219_MOESM1_ESM.doc]

**Cover letter**

Dear Editors:

We would like to submit the enclosed manuscript entitled “Quality of Life among People with Eye Cancer: A Systematic Review from 2012 to 2022”, which we wish to be considered for publication in “Health and Quality of Life Outcomes”. No conflict of interest exits in the submission of this manuscript, and manuscript is approved by all authors for publication. I would like to declare on behalf of my co-authors that the work described was original research that has not been published previously, and not under consideration for publication elsewhere, in whole or in part. All the authors listed have approved the manuscript that is enclosed.

In this work, we evaluated Quality of Life among People with Eye Cancer: A Systematic Review from 2012 to 2022.It is the first review concentrate on the QOL on both RB and UM patients, and find the gap on this field. I hope this paper is suitable for“Health and Quality of Life Outcomes”.

The following is a list of possible reviewers for your consideration:

1)Name A E-mail: 1641694882@qq.com

2)Name B E-mail: huangyonghui@163.com

We deeply appreciate your consideration of our manuscript, and we look forward to receiving comments from the reviewers. If you have any queries, please do not hesitate to contact me at the address below.

Thank you and best regards.

Yours sincerely,

Yonghui Huang

Corresponding author:

Name: Yonghui Huang

E-mail: 1641694882@qq.com
